# Mesenchymal stem cell-derived exosomes as a promising cell-free therapy for knee osteoarthritis

**DOI:** 10.3389/fbioe.2024.1309946

**Published:** 2024-01-16

**Authors:** Danni Luo, Hao Zhu, Song Li, Zhenggang Wang, Jun Xiao

**Affiliations:** Department of Orthopedics, Tongji Hospital, Tongji Medical College, Huazhong University of Science and Technology, Wuhan, China

**Keywords:** exosomes, osteoarthritis, mesenchymal stem cells, tissue engineering, cartilage regeneration

## Abstract

Osteoarthritis (OA), as a degenerative disease, leads to high socioeconomic burdens and disability rates. The knee joint is typically the most affected and is characterized by progressive destruction of articular cartilage, subchondral bone remodeling, osteophyte formation and synovial inflammation. The current management of OA mainly focuses on symptomatic relief and does not help to slow down the advancement of disease. Recently, mesenchymal stem cells (MSCs) and their exosomes have garnered significant attention in regenerative therapy and tissue engineering areas. Preclinical studies have demonstrated that MSC-derived exosomes (MSC-Exos), as bioactive factor carriers, have promising results in cell-free therapy of OA. This study reviewed the application of various MSC-Exos for the OA treatment, along with exploring the potential underlying mechanisms. Moreover, current strategies and future perspectives for the utilization of engineered MSC-Exos, alongside their associated challenges, were also discussed.

## 1 Introduction

Osteoarthritis (OA) is the most common chronic degenerative joint disease, marked by gradual deterioration of articular cartilage, subchondral bone remodeling, osteophyte formation and synovial inflammation (Jiang, 2022). With an ageing population, OA is emerging as a major health issue, impacting over 300 million people worldwide, more than 40% of whom are over the age of 70 (Hunter and Bierma-Zeinstra, 2019; Kolasinski et al., 2020). An increasing number of research have indicated that articular cartilage and subchondral bone form a functional unit that has a coherent and reciprocal effect on the development of OA (Hu et al., 2021).

Articular cartilage consists of chondrocytes and extracellular matrix (ECM). As the primary cellular constituents of cartilage, chondrocytes play a fundamental role in synthesizing and maintaining ECM to preserve the structural integrity of articular cartilage (Sophia Fox et al., 2009). Specifically, chondrocytes secrete various ECM components, including lubricin, glycoproteins and type II collagen (COL2) fibers to maintain a stable environment within articular cartilage (Gilbert et al., 2021). Chondrocyte function is intricately regulated by multiple factors. Physiological loading from joint movement and exercise is beneficial, stimulating chondrocytes to maintain cartilage integrity (Deng et al., 2023). However, abnormal mechanical loading can lead to cartilage degeneration. Additionally, inflammatory mediators like interleukin-1 (IL-1) and tumor necrosis factor-alpha (TNF-α) negatively impact chondrocyte function and accelerate cartilage degradation (Schuerwegh et al., 2003). During the development of OA, the homeostasis within the articular cartilage is disrupted. Chondrocytes undergo hypertrophic changes and abnormally secrete multiple cartilage matrix-degrading enzymes, such as a disintegrin and metalloproteinase with thrombospondin motifs 5 (ADAMTS5), matrix metalloproteinase-3 (MMP-3) and matrix metalloproteinase-13 (MMP-13) (Cho et al., 2021). These matrix-degrading enzymes sequentially degrade the cartilage matrix, leading to articular cartilage degeneration. However, cartilage has a limited regenerative capacity compared to other tissues such as skin or blood vessels due to its avascular nature and low cell turnover rate (Gilbert et al., 2021). Cartilage regeneration is a complex process involving chondrocytes proliferation and differentiation. When the injury occurs, mesenchymal stem/stromal cells (MSCs) can be recruited to the specific site, which have the potential to differentiate into chondrocytes to replace damaged tissue and are responsible for producing the ECM of cartilage (Jablonski et al., 2019).

**FIGURE 1:**
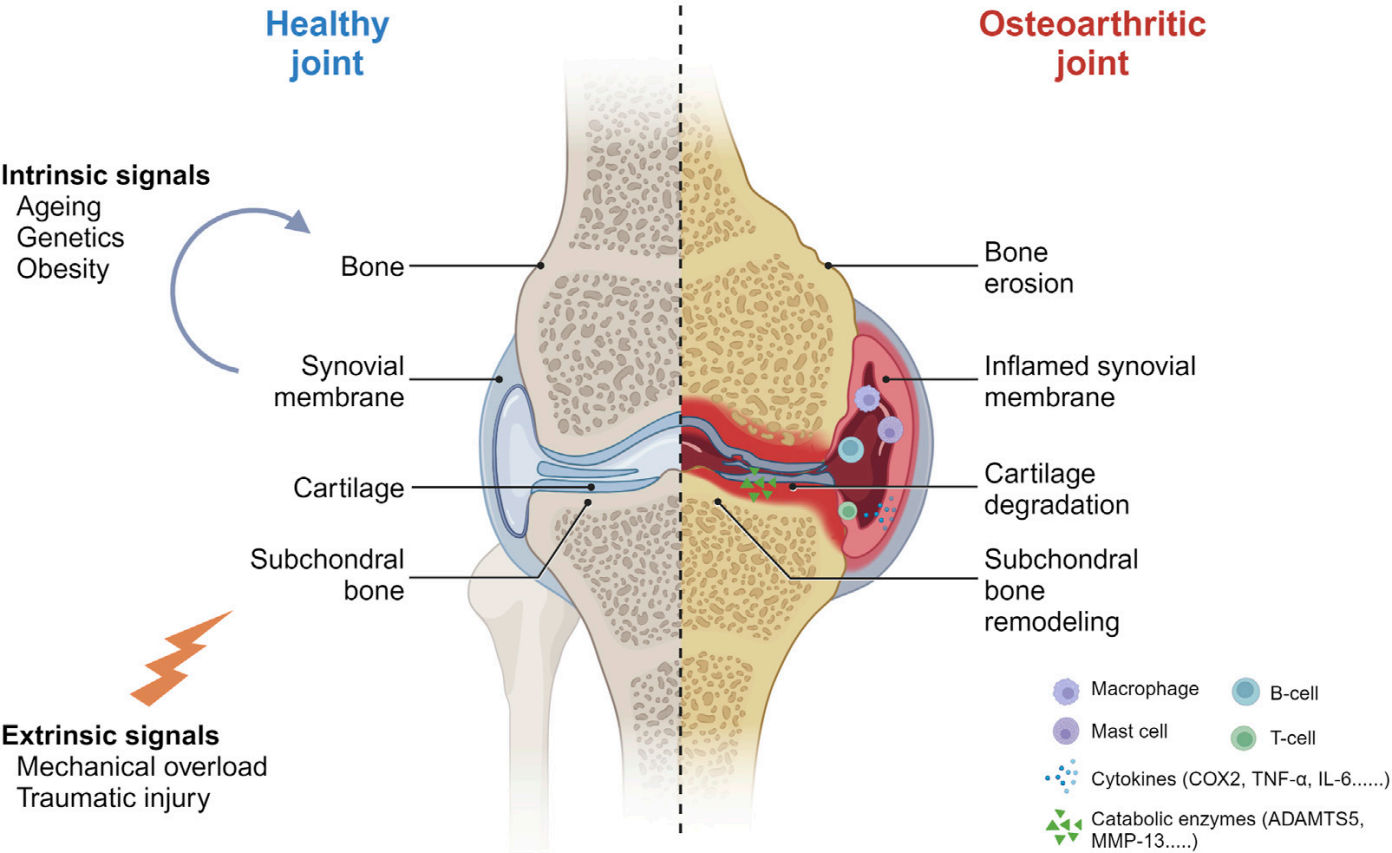
The pathobiological network of osteoarthritis. Ageing, genetics, obesity, mechanical overload and traumatic injury are reported to be mainly risk factors that may improve the susceptibility to OA. OA comes with various symptoms like cartilage degradation and subchondral bone remodeling. Numerous cytokines and catabolic enzymes are associated during OA progression (Created with BioRender.com).

Subchondral bone supplies mechanical support to cartilage and undergoes dynamic remodeling to adapt to microenvironmental changes (Lu et al., 2023). Compared to articular cartilage, subchondral bone exhibits a greater capacity in response to surrounding mechanical stress (Hu et al., 2021). In early-stage OA, accelerated bone resorption and reduced subchondral bone plate thickness precede obvious cartilage degeneration (Kazemi and Williams, 2021; Hu et al., 2022). Subsequently, cartilage destruction occurs primarily in areas where the subchondral bone plate thickness is decreased. As OA progresses, subchondral bone resorption rate is significantly reduced, resulting in uncoupled remodeling of subchondral bone, which is manifested by an abnormal thickening of the subchondral bone growth plates (Kazemi and Williams, 2021). This is also one of the significant pathological signs of the late stage of OA (Figure 1).

Currently, OA treatment can be categorized into two main groups. One is early medication, including non-steroidal anti-inflammatory drugs (NSAIDs), which are used primarily to relieve symptoms, or glucosamine, hyaluronic acid and chondroitin sulphate, which help protect cartilage. However, drug treatment merely decelerates the progression of OA and may augment the probability of adversities towards the gastrointestinal tract and cardiovascular system (Richette et al., 2015). Surgical treatment, such as subchondral bone microfracture, autologous chondrocyte implantation and knee arthroplasty, is considered when conservative treatment is unsatisfactory (Rahmani Del Bakhshayesh et al., 2020). Nonetheless, it is not only imposing a heavy economic burden on individuals but also to their families and even the whole society. Therefore, intervening early in the disease process and enhancing damaged cartilage reconstruction are currently the primary goals of OA treatment.

Over the past decade, cell-based therapies have rapidly emerged as a promising approach to articular cartilage repair. Numerous preclinical studies have shown that injecting MSCs into joint cavity can enhance cartilage regeneration and reduce synovial inflammation to alleviate OA progression (Desando et al., 2013). Although a systematic review reported that MSC-based therapy could significantly reduce pain symptoms and repair joint function (Wei et al., 2021), there are still some challenges to clinical implementation. For example, potential pro-tumorigenic effects, lack of standardized cell production and ethical audit, which have led researchers to investigate alternative approaches in the field of MSC-based biological tissue engineering (Lukomska et al., 2019). Recently, a growing number of evidence supports that MSC-derived extracellular vesicles (MSC-EVs) play a crucial role in intercellular communication and retain valuable properties of parental cells (Thakur et al., 2022). Compared to cell-based therapy, EVs show great advantages such as low immunogenicity, good stability, no ethical controversy, easy storage and direct fusion with target cells (Zhou et al., 2022). These attributes make EV-based therapy as a potential substitute for MSC-based cell therapy.

EVs comprise various subtypes such as microvesicles, apoptotic bodies and exosomes (Exos), each of which plays a unique role in several biological processes (Srinivasan and Sundar, 2021). Among them, exosomes have received more attention than other EVs due to their outstanding performance (Samanta et al., 2018; Gurunathan et al., 2019). Exosomes are membrane-bound vesicles characterized by nanoscale dimensions (typically in the range of 30–150 nm). They can be isolated from various bodily fluids, including blood, plasma and saliva, and derived from a diverse range of cell varieties such as fibroblasts, immune cells, tumour cells, chondrocytes and MSCs (Zhu et al., 2020). Exosomes possess the capacity to deliver a wide range of bioactive molecules, making them a potent tool for intercellular communication and therapeutic applications. Importantly, exosomes play a critical role in various physiological and pathological processes, including maintaining cellular homeostasis, regulating apoptosis and modulating inflammation (Kalluri and LeBleu, 2020; Kim, 2022).

Recently, numerous investigations have shown that MSC-derived exosomes (MSC-Exos) can be effectively used for tissue repair and immunomodulation (Wang et al., 2018; Yu et al., 2022). In addition, several systematic reviews have mentioned that MSC-Exos, as a potential strategy for OA, can attenuate OA progression by mitigating cartilage degradation and enhancing chondrocyte phenotype (To et al., 2020; Zhang et al., 2021; Tan et al., 2021). A completed clinical trial reported that 6 months after the injection of 2 mL ExoFlo (a BM-MSC-Exos product), pain was significantly reduced and joint function improved, indicating BMMSC-Exos was safe and effective for the treatment of OA (Dordevic M., 2020). In this review, we summarized the applications of various MSC-Exos for OA treatment and the underlying mechanisms. Moreover, current methods and future perspectives for the utilization of engineered MSC-Exos, alongside their associated challenges, were also discussed.

## 2 The potential mechanisms of MSC-Exos for OA treatment

**FIGURE 2:**
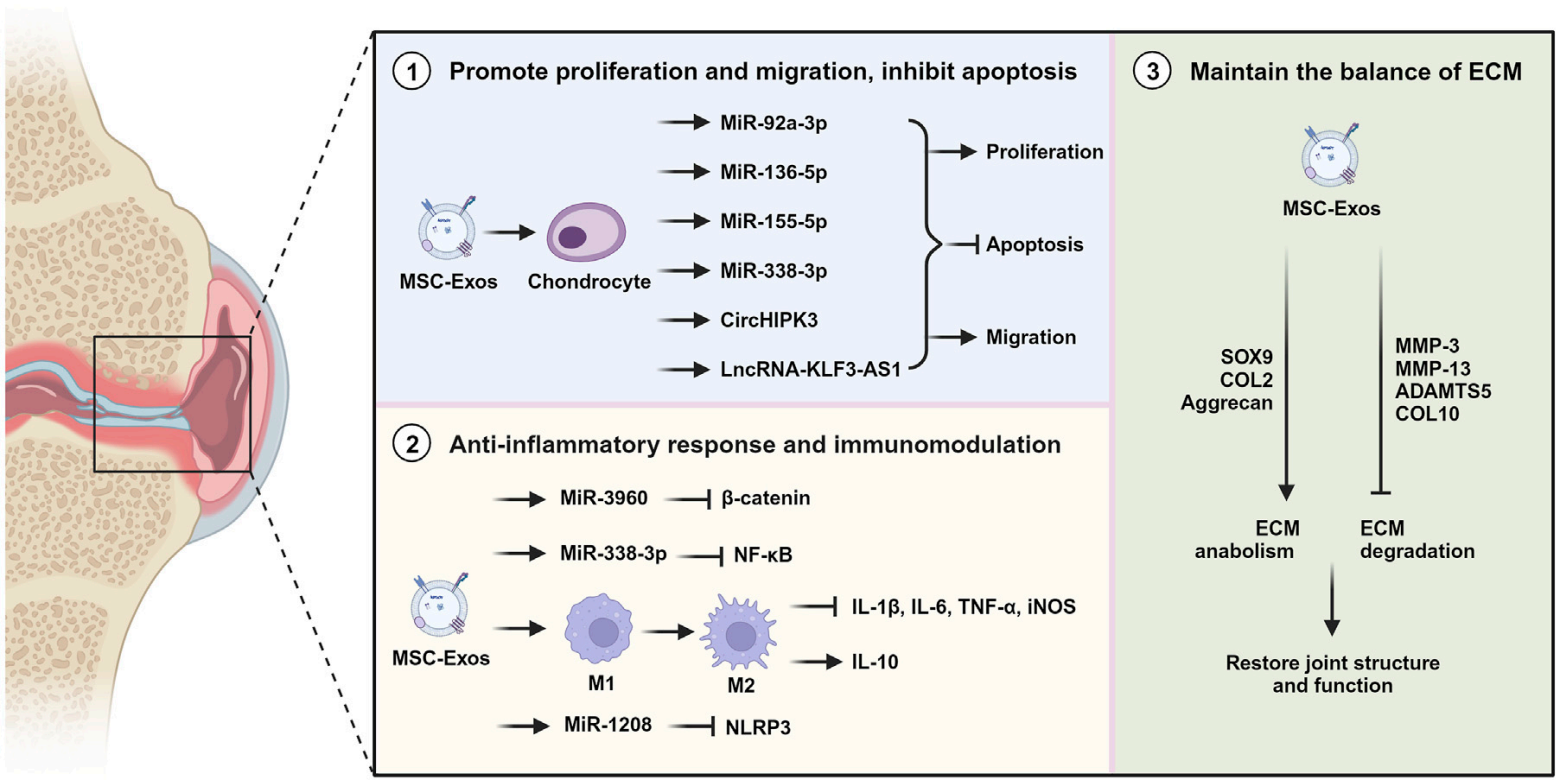
The potential mechanisms of MSC-Exos for OA treatment. MSC-Exos mitigate OA through stimulating cell proliferation, preventing apoptosis, triggering anti-inflammatory responses, modulating the immune system, and preserving the ECM equilibrium (Created with BioRender.com).

MSC-Exos, serving as vital messengers for cartilage regeneration and intercellular communication, have shown remarkable potential to mitigate the progression of OA by modulating various cellular processes (Kim et al., 2020; Wu et al., 2022). They were reported to promote chondrocyte proliferation, inhibit chondrocyte apoptosis, reduce pro-inflammatory cytokines, modulate immune responses and redeposit cartilage matrix (Figure 2). Exosomes derived from various types of MSCs were used in OA-related cell and animal experiments (Table 1). And the efficacy of exosomes is also influenced by different sources of tissues (Li et al., 2021; Wang et al., 2020).

**TABLE 1:** The impact of exosomes sourced from various types of mesenchymal stem cells on OA.

Exosome source	Cargo	Biological effect	References
BM-MSCs	MiR-92a-3p	Promote proliferation while suppress degradation of cartilage in OA model	Mao et al. (2018)
	MiR-136-5p	Promote chondrocyte migration, reduce the degeneration of cartilage extracellular matrix in OA model	Chen et al. (2020)
	MiR-320c	Promote osteoarthritis chondrocyte proliferation, downregulate MMP-13 and upregulate SOX9 expression	Sun et al. (2019)
	MiR-3960	Decrease the degradation of ECM and reduce the ratio of apoptosis in chondrocytes	Ye et al. (2022)
	MiR-125a-5p	Alleviate chondrocytes degeneration while promote ECM secretion	Xia et al. (2021)
	MiR-361-5p	Mitigate the damage of chondrocytes	Tao et al. (2021b)
	CircHIPK3	Induce migration and proliferation, inhibit apoptosis of chondrocytes	Li et al. (2021b)
	LncRNA NEAT1	Activate the proliferation and autophagy of chondrocytes	Zhang and Jin (2022)
	LncRNA LYRM4	Regulate chondrocyte growth and reduce inflammation in OA	Wang et al. (2021b)
	N.D.	Increase the repair of cartilage and the viability of chondrocytes	Yang et al. (2022)
UC-MSCs	LncRNA H19	Decrease pain level in early stage of OA via enhancing chondrocyte proliferation and matrix synthesis	Yan et al. (2021)
	MiR-1208	Suppress cartilage ECM degradation via decreasing level of pro-inflammatory factors	Zhou et al. (2022b)
	MiR-100-5p	Inhibit apoptosis and ROS production in chondrocytes	Li et al. (2021d)
	MiRNAs	Promote M2 macrophage polarization, lessen the progression of ACLT-induced OA	Li et al. (2022c)
S-MSCs	MiR-140-5p	Promote chondrocyte proliferation and migration, delay early-stage OA progression	Tao et al. (2017)
	MiR-26a-5p	Inhibit apoptosis and inflammation, ameliorate cartilage injury of OA	Lu et al. (2021)
	MiR-155-5p	Promote proliferation, inhibit apoptosis and regulate secretion of ECM	Wang et al. (2021b)
	MiR-129-5p	Decline chondrocyte apoptosis and inflammatory response	Qiu et al. (2021)
	CircRNA3503	Preserve the equilibrium of ECM in chondrocytes	Tao et al. (2021a)
	N.D.	Inhibit ECM degradation	Duan et al. (2021)
AT-MSCs	DKK-1	Promote chondrogenesis and chondrocyte redifferentiation	Gorgun et al. (2021)
	MiR-338-3p	Stimulate cell proliferation and inhibit cell apoptosis	Li et al. (2022d)
	N.D.	Attenuate inflammatory micro-environment	Cavallo et al. (2021)
ESC-MSCs	N.D.	Improve the effect on cartilage repair via cell proliferation and apoptosis	Zhang et al. (2018)
	N.D.	Induce cartilage repair *in vivo*	Zhang et al. (2016)
	N.D.	Maintain chondrocyte phenotype and alleviate cartilage destruction *in vivo*	Wang et al. (2017)
IPFP-MSCs	MiR-100-5p	Suppress cartilage apoptosis, promote anabolism and prevent cartilage injury in cell and animal experiments	Wu et al. (2019)
Urine MSCs	MiR-140-5p	Increase ECM secretion and enhance the ability of cell proliferation	Liu et al. (2022)
	MiR-26a-5p	Promote cell migration and proliferation	Wan et al. (2022)

MSC, mesenchymal stem cells; OA, osteoarthritis; N.D., no data; BM-MSCs, bone marrow derived MSCs; UC-MSCs, umbilical cord derived MSCs; S-MSCs, synovial MSCs; IPFP-MSCs, infrapatellar fat pad derived MSCs; AT-MSCs, adipose tissue derived MSCs; ESC-MSCs, embryonic stem cell derived MSCs; ACLT, transection of the anterior cruciate ligament; DKK1, Dickkopf-1; MMP-13, matrix metallopeptidase-13; SOX9, (sex determining region Y)-box 9; COX2, cyclooxygenase-2; ECM, extracellular matrix; ROS, reactive oxygen species.

### 2.1 Effect on cartilage repair

Cartilage faces challenges in self-repair due to its avascular nature and limited exchange of signaling molecules, oxygen and nutrients (Carballo et al., 2017). MSC-Exos, which target biological processes such as proliferation and apoptosis of chondrocytes, exhibit great capability for the treatment of OA (Xiang et al., 2022).

A variety of MSC-Exos have been employed to enhance chondrocyte proliferation and migration, thereby promoting cartilage restoration (Charlier et al., 2016). Zhu et al. reported that exosomes derived from induced pluripotent stem cell-derived MSCs (iMSC-Exos) and synovial MSCs (SMSC-Exos) could enhance the proliferation and migration of chondrocytes, however iMSC-Exos showed a superior effect compared to SMSC-Exos (Zhu et al., 2017). One study found that exosomes derived from bone marrow-derived MSCs (BMMSC-Exos) affected chondrocyte viability, proliferation and migration by improving mitochondrial activity (Yang et al., 2022). In addition, Li et al. proved that MSC-EVs containing circHIPK3 could enhance chondrocyte proliferation and simultaneously suppress chondrocyte apoptosis via combining with miR-124-3p and then targeting the gene MYH9 (Li et al., 2021b). Furthermore, BMMSC-Exos, delivering the lncRNA LYRM4-AS1, modulated the viability of IL-1β-induced chondrocytes via the LYRM4-AS1/GRPR/miR-6515-5p axis (Wang et al., 2021). Another study confirmed that human umbilical cord-derived MSCs exosomes (UCMSC-Exos) could effectively promote chondrocytes proliferation and migration (Li et al., 2022). It was reported that MSC-Exos derived from embryonic stem cell (ESCMSC-Exos) promoted the proliferation and migration of chondrocytes without affecting matrix synthesis through adenosine-mediated activation of AKT and ERK signaling pathways (Zhang et al., 2018). Additionally, an *in vitro* study verified that human umbilical cord Wharton’s jelly MSCs-derived exosomes (WJMSC-Exos) can increase chondrocyte proliferation in a dose-dependent manner (Jiang et al., 2021).

MSC-Exos have also been illustrated to inhibit chondrocyte apoptosis. The apoptosis of chondrocytes is associated with many signaling pathways, particularly those involving phosphorylation. Qi et al. noted that BMMSC-Exos promoted Akt phosphorylation while inhibited ERK and p38 phosphorylation, consequently suppressing mitochondrial-induced apoptosis in chondrocytes Qi et al. (2019). Besides, Jin et al. demonstrated that BMMSC-Exos containing lncRNA MEG-3 could mitigate IL-1β-induced chondrocyte senescence and apoptosis, effectively inhibiting OA progression Jin et al. (2021). Studies have shown that MSC-Exos are capable to activate the mTOR pathway, which promotes autophagy to inhibit apoptosis and improve chondrocyte performance (Shen et al., 2017; Wu et al., 2019). The ratio of the anti-apoptosis gene Bcl-2 to the apoptosis gene Bax can influence whether or not chondrocytes undergo apoptosis (KARALIOTAS et al., 2015). It was reported that ESCMSC-Exos elevated the levels of Survivin and Bcl-2 expression while reduced the proportion of cleaved caspase-3-positive apoptotic cells *in vivo* (Zhang et al., 2018). Additionally, a study demonstrated that UCMSC-Exos, including miR-100-5p, could directly target NOX4 to inhibit ROS production and apoptosis induced by cyclic strain in chondrocytes (Li et al., 2021). Lu et al. verified that SMSC-EVs containing miR-26a-5p mitigated cartilage damage via inhibiting cartilage apoptosis and directly targeting the PTEN gene *in vivo* Lu et al. (2021).

### 2.2 Anti-inflammatory response and immunomodulation

The progression of OA is positively correlated with the degree of inflammatory infiltration. Inflammatory cytokines are secreted, leading to induced immune responses that play a role in OA pathogenesis and progression. Several studies indicate that MSC-Exos possess the ability to regulate inflammatory responses by lowering concentrations of pro-inflammatory factors and promoting secretions of anti-inflammatory cytokines (Hassanzadeh et al., 2023).

Macrophages and synovial cells are closely associated with the initiation and progression of inflammation (Oishi and Manabe, 2018). Peng et al. found that MSC-Exos could prevent macrophage ferroptosis through the GOT1/CCR2/Nrf2/HO-1 signaling pathway and rescue cartilage injury in OA Peng et al. (2023). Shifting of synovial macrophages from a pro-inflammatory to an anti-inflammatory phenotype has the potential to significantly impact the development of the intra-articular microenvironment (Wang and He, 2022). It was reported that WJMSC-EVs effectively promoted the polarization of macrophages towards an M2 phenotype, thereby reducing the inflammatory response (Joo et al., 2021). In addition, Zhang et al. showed that ESCMSC-Exos induced a large number of M2 macrophages to infiltrate into the synovial fluid *in vivo* Zhang et al. (2018). Another study demonstrated that microRNAs (miRNAs) in human amniotic membrane-derived MSC-EVs, such as miR-24-3p, miR-222-3p, miR-146a-5p, miR-34a-5p and miR-181a-5p, could influence macrophage activation states, promote M2 macrophage polarization, and stimulate cartilage regeneration (Ragni et al., 2021). Furthermore, UCMSC-Exos, containing miR-100-5p, miR-let-7a-5p, miR-122-5p, miR-486-5p and miR-148a-3p, facilitate macrophage polarization towards an M2 phenotype and attenuate the deterioration of ACLT-induced OA (Li et al., 2022).

During the progression of OA, several pro-inflammatory factors, including tumor necrosis factor-alpha (TNF-α), interleukin-1 beta (IL-1β) and IL-6, are released to accelerate the degeneration of cartilage (Pourakbari et al., 2019; Lee et al., 2020). IL-4, IL-10 and transforming growth factor-beta (TGF-β), acting as anti-inflammatory cytokines, are secreted by M2 macrophages to repair the cartilage (Fernandes et al., 2020). A study showed BMMSC-Exos regulated the levels of IL-6 and TNF-α in chondrocytes and tissues (Jiang et al., 2021). Moreover, exosomal miR-9-5p derived from BMMSC was proved to inhibit SDC1 expression, further decreased IL-1 and TNF-α in ACLT-induced OA (Jin et al., 2020). It was proved that adipose tissue-derived MSCs exosomes (ATMSC-Exos), containing miR-145 and miR-221, upregulated the level of IL-10 while downregulated the expression of TNF-α and IL-6 (Zhao et al., 2020). Besides, it was shown that SMSC-derived exosomal miR-129-5p could decrease the inflammation in IL-1β-induced OA by inhibiting HMGB1 release (Qiu et al., 2021).

### 2.3 Maintain the balance of ECM

The gradual cartilage matrix deterioration is pivotal in OA pathology, triggering the breakdown of joint structure and consequent damage. To promote the redeposition of cartilage ECM and maintain cartilage integrity, it is essential to activate reparative responses in chondrocytes and enhance the expression of genes related to synthetic metabolism (Heard et al., 2015). The investigation into how MSC-Exos maintain ECM balance has been conducted.

Several studies indicated that MSC-Exos could downregulate ADAMTS-5, MMP-3 and MMP-13 expression, while upregulate the levels of tissue inhibitors of metalloproteinases (TIMPs), COL2, glycosaminoglycans (GAGs), and sex-determining region Y-Box 9 (SOX9) (Lozito and Tuan, 2011; Lozito et al., 2014). Cosenza et al. and Vonk et al. reported that BMMSC-Exos could promote the production of proteoglycan, COL2 and aggrecan, while inhibiting the expression of MMP-13 and ADAMTS5 and the activity of collagenase Cosenza et al. (2017), Vonk et al. (2018). Besides, ATMSC-Exos were demonstrated to effectively improve COL2 expression while reducing ADAMTS-5 and MMP-1, -3, -13 expression in chondrocytes, thereby attenuating cartilage matrix degradation in the monosodium iodoacetate (MIA)-induced OA model (Woo et al., 2020). Furthermore, Jammes et al. found that equine BMMSC-derived exosomes induced a greater improvement in hyaline-like matrix neosynthesis by modulating collagen levels, increasing PCNA, and decreasing Htra1 synthesis Jammes et al. (2023).

Exosomal RNAs have shown great potential in promoting cartilage ECM repair. It was reported that BMMSC-derived exosomal miR-320c increased chondrocyte proliferation by increasing the expression of SOX9 and decreasing MMP-13 levels (Sun et al., 2019). Another study showed that BMMSC-Exos could upregulate the levels of COL2 and aggrecan alongside downregulate ADAMTS-5 and MMP-13 expression by encapsulating miR-3960 (Ye et al., 2022). Moreover, BMMSC-derived exosomal miR-125a-5p was demonstrated to alleviate chondrocyte ECM degradation via inhibiting E2F2 in post-traumatic OA (Xia et al., 2021). Wang et al. found that SMSC-Exos containing miR-155-5p enhanced the secretion of ECM in chondrocytes by negatively regulating Runx2 expression to prevent OA Wang et al. (2021a). Zhou et al. showed that UCMSC-Exos suppressed the degradation of cartilage ECM in OA mouse models via miR-1208, which targeting METTL3 to decrease NLRP3 mRNA methylation in macrophages Zhou et al. (2022a).

## 3 Engineering strategies of MSC-Exos for OA treatment

**FIGURE 3:**
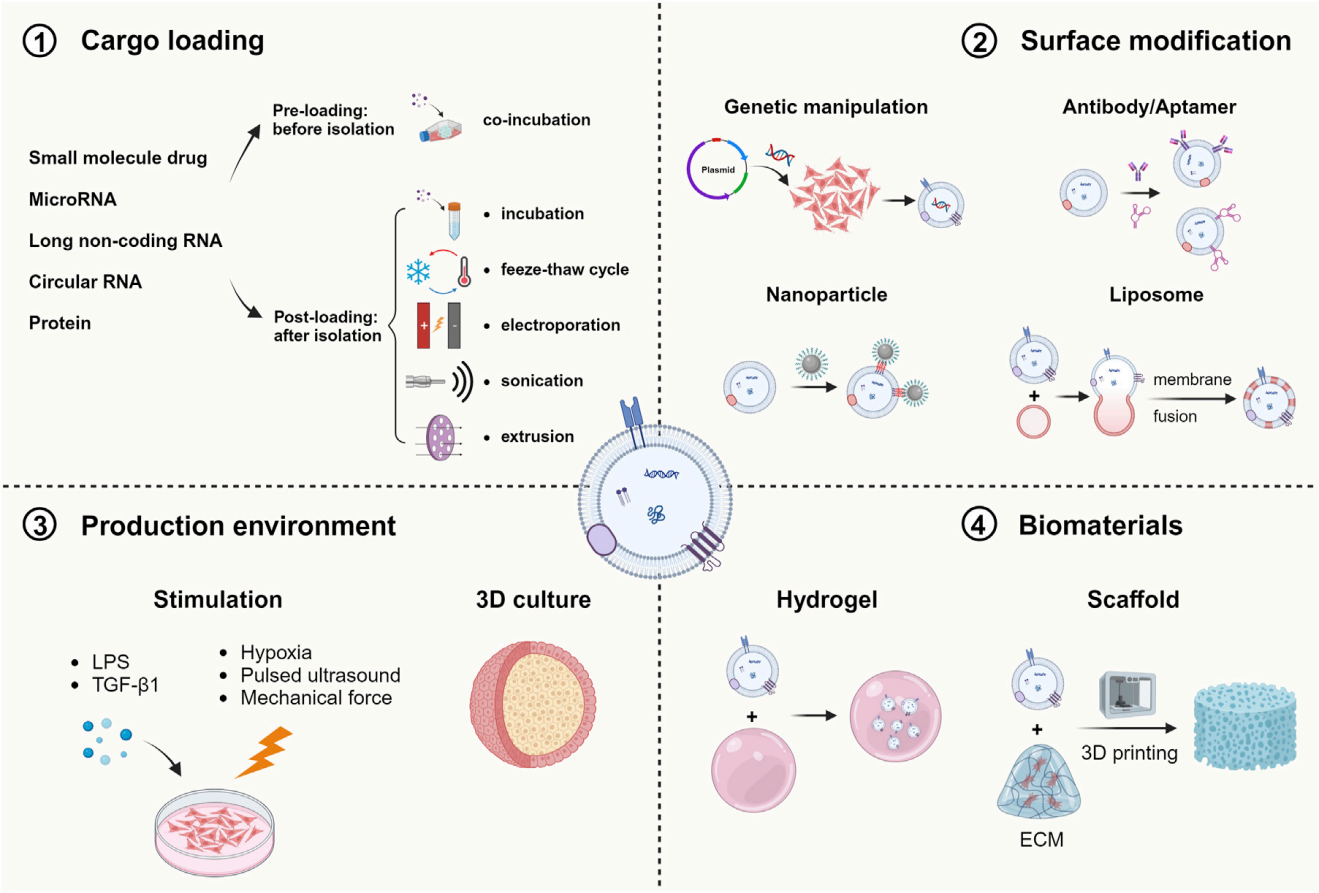
Engineering Strategies of MSC-Exos for OA treatment. Various methods have been utilized to engineer ESC-Exos in order to elevate the therapeutic effect via cargo loading, surface modification, changing the production environment and combining with biomaterials (Created with BioRender.com).

Despite natural exosomes have great potential for cartilage tissue repair, they still come with some limitations such as low yield, circulatory stability and inadequate targeting ability, making them insufficient for disease treatment (Kimiz-Gebologlu and Oncel, 2022). To overcome these challenges and advance the clinical application of exosome therapy, various engineering approaches have been developed, including cargo loading, surface modification, changing the production environment and combination of biomaterials, focusing on both parent cells and exosomes (Figure 3).

### 3.1 Cargo loading strategies of MSC-Exos

Two main strategies for loading cargo into exosomes are pre-loading and post-loading (Xu et al., 2023). Pre-loading entails loading cargo into parent cells before isolating exosomes, resulting in the secretion of exosomes already loaded with cargo. However, post-loading involves loading cargo directly into exosomes using passive or active techniques after they have been isolated (Elsharkasy et al., 2020; Soekmadji et al., 2020).

The enrichment of therapeutic molecules in MSC-Exos is mainly accomplished through the overexpression of various non-coding RNAs, including miRNAs, long non-coding RNAs (lncRNAs), circular RNAs (circRNAs) and others. Among these, there is extensive evidence supports that miRNAs can promote Exos-mediated the regeneration of cartilage (Foo et al., 2021). One study showed that miR-92a-3p-overexpressing BMMSC-Exos promoted cartilage proliferation and reduced cartilage matrix synthesis by targeting WNT5A and inhibiting WNT signaling pathway (Mao et al., 2018). Zheng et al. reported that miR-212-5p-overexpressing SMSC-Exos reduced the degeneration, degradation and inflammation processes by targeting ELF3 in IL-1β-induced chondrocytes Zheng et al. (2022). Another study demonstrated that exosomes derived from miR-140-5p-overexpressing SMSCs enhanced cartilage tissue repair and mitigated OA progression in an animal model via the WNT signaling pathway (Tao et al., 2017). Wen et al. showed that exosomes derived from lncRNAs KLF3-AS1-overexpressing MSCs were involved in suppressing apoptosis and autophagy of chondrocytes via PI3K/Akt/mTOR signaling pathway (Wen et al., 2022). In addition, Li et al. reported that circHIPK3 was observed to directly sponge miR-124-3p and subsequently enhance the MYH9 expression, contributing to promoting chondrocyte proliferation while suppressing chondrocyte apoptosis mediated by MSC-Exos Li et al. (2021b). Furthermore, SMSC-derived exosomal circRNA3503, acting as a sponge for hsa-let-7b-3p and hsa-miR-181c-3p, ameliorated chondrocyte apoptosis induced by inflammation and regulated the balance of ECM synthesis and degradation (Tao et al., 2021). Shuai et al. showed that exosomal CircRNA0008365 enhanced the expression of SOX9 by sponging miR-338-3p, leading to inhibition of chondrocyte apoptosis and ECM degradation in OA Shuai et al. (2022).

Small molecule drugs and proteins can also be encapsulated using vairous methods. In a sheep OA model, MSC-Exos loaded with TGF-β3 and bone morphogenetic protein-6 (BMP-6) increased cartilage repair and chondrogenesis (Ude et al., 2018; Yoo et al., 2022). Thomas et al. revealed that exosomes loaded with WNT3a successfully initiated WNT signaling in cartilage, contributing to osteochondral defects repair in an OA model (Thomas et al., 2021). Besides, Qiu et al. showed that MSC-Exos loading with curcumin inhibited the apoptosis of OA cells via miR-143/ROCK1/TLR9 and miR-124/NF-kB signaling pathways Qiu et al. (2020).

### 3.2 Surface modification strategies of MSC-Exos

Enhancing the targeting capacity of exosomes by incorporating specific ligands on their surface enables the precise delivery of therapeutic cargo to the disease site, which is a critical factor for effective treatment of OA. Zhao et al. found that chondrocyte-binding peptide (CAP) binding subcutaneous fat MSC-derived exosomes could particularly send miR-199a-3p into targeting cells and deep articular tissues, which showed great effect on OA progression (Zhao et al., 2023). And CAP-exosomes had the potential to deliver miR-140 to chondrocytes and deep cartilage region *in vitro* and *in vivo*, alleviating OA progression by inhibiting cartilage-degrading proteases (Liang et al., 2020). Researchers found that the MSC-binding peptide E7 could be fused with exosomal membrane protein Lamp2b to construct functional exosomes (E7-SMSC-Exos) with SMSC targeting capability, which could efficiently induce cartilage differentiation when further combined with KGN (Xu et al., 2021). Another study showed that ATMSC-Exos binding with chitosan oligosaccharides (COS) facilitated regeneration of injury cartilage and protect chondrocytes from apoptosis by regulating vital pathways such as WNT and MAPK in OA progression (Li et al., 2021c). Nanoparticles combined with exosomes can have positive effects on functions. Li et al. reported that CD90-positive SMSC-Exos-coated nanoparticle could bind to injured chondrocytes, promote chondrocyte regeneration, and influence M2 macrophage polarization in a rat OA model Li et al. (2022c). In addition, by fusing CAP to Lamp2b on exosomal surfaces and subsequently merging with liposomes, Liang et al. found that the hybrid CAP-Exos could successfully deliver CRISPR/Cas9 sgMMP-13 plasmids to silence MMP-13 expression, thereby mitigating the hydrolytic degradation of ECM proteins in the deep regions of damaged cartilage in a rat model Liang et al. (2022).

### 3.3 Production environment of MSC-Exos

In addition to directly increasing the content of therapeutic molecules, altering the environment of production for MSC-Exos also presents a favorable engineering strategy.

An effective method for generating MSC-Exos in significant amounts is by expanding MSCs, which can be accomplished by enlarging the available surface area for cellular proliferation (Cheng et al., 2022). Rocha and others showed that MSC-Exos cultured using a three-dimensional (3D) approach generated a higher quantity of exosomes in comparison to the traditional two-dimensional (2D) method, illustrating the advantage of the 3D method for scaling up exosome production Rocha et al. (2019). Further study found that 3D-Exos exhibited a 7.5-time higher yield compared to 2D-Exos. In addition, UCMSC-Exos cultured in a 3D environment demonstrated a notably enhanced therapeutic efficacy than their 2D counterparts (Yan and Wu, 2020). Furthermore, Dias et al. found that a poly (ethylene glycol) (PEG)-based microcarrier could enhance the adhesion and expansion capabilities of human MSCs Dias et al. (2017). Another study showed that decellularized extracellular matrix (dECM) could provide a better microenvironment for MSC expansion, and significantly increased miR-3473b levels in dECM-BMMSC-Exos, which had a better ability to regenerate cartilage than BMMSC-Exos *in vivo* (Zhang et al., 2023).

To adapt to the environment, cells can transmit stress-related information by regulating the release of exosomes. Previous studies showed that the expression of miR-135b in BMMSC-Exos could be enhanced by TGF-β1 stimulation, leading to a decrease in the expression of Sp1, promoting the proliferation of chondrocytes (Wang et al., 2018). Rong et al. reported that exosomes derived from HIF-1α-induced hypoxic BMMSCs enhanced the chondrocyte proliferation while suppressed chondrocyte apoptosis compared to normal BMMSC-Exos Rong et al. (2021). Additionally, hypoxia-treated ATMSC-Exos increased collagen and proteoglycan expression in cartilage and normalized uncoupled bone remodeling in subchondral bone compared to the normal ADSC-Exo group in a murine OA model (Zhao et al., 2023). Chang et al. found that hypoxia-ATMSC-Exos improved articular chondrocyte function, alleviated articular chondrocyte inflammation and suppressed the OA progression in cell and animal experiments Chang et al. (2023). Another study demonstrated that mechanical stimulation from a rotary cell culture system could expand the exosome yield, and then enhance the repair of cartilage defect by up-regulating LncRNA H19 in UCMSC-Exos (Yan et al., 2021). Furthermore, Liao et al. showed that BMMSC-Exos treated with low-intensity pulsed ultrasound inhibited inflammation and further enhanced chondrocyte proliferation and ECM synthesis Liao et al. (2021).

### 3.4 Biomaterials for MSC-Exos retention and delivery

In OA treatment, the prevailing approach for exosome delivery is intra-articular injection (Bousnaki et al., 2020). An increasing number of researches are focusing on combing exosomes with biomaterials to prolong retention time and improve therapeutic effect.

Hydrogel is a favourable biomaterial for cartilage tissue engineering applications due to its injectability and cross-linking capability under UV exposure. Pang et al. reported that gelatin methacryloyl hydrogels (GelMA) facilitated the prolonged release of MSC-Exos and significantly enhanced their therapeutic impact on OA Pang et al. (2023). Wan et al. applied photocrosslinking spherical gelatin methacryloyl hydrogel to act as injectable carriers for LRRK2-IN-1-loaded exosomes Wan et al. (2023). The results indicated that engineered BMMSC-Exos had a superior effect on cartilage repair *in vivo*. In a previous investigation, researchers explored the application of an adhesive, injectable hydrogel inspired by mussels, which incorporated BMMSC-Exos. They were utilized to promote regeneration of cartilage defects and the remodeling of the ECM (Zhang et al., 2021).

Exosomes can collaborate with bioactive scaffolds, especially ECM-derived scaffolds, to improve capabilities of promoting cartilage repair (Cheng et al., 2022). Jiang et al. found the regenerative effect of WJMSC-Exos was amplified by the incorporation of the acellular cartilage ECM (ACECM) scaffold in a rabbit model Jiang et al. (2021a). Mechanically, the ACECM scaffold provided a cartilage-like microenvironment that facilitated the attachment of local cells (Sun et al., 2018). Using desktop-stereolithography technology, Chen et al. reported that they designed an innovative 3D-printed cartilage ECM/GelMA/exosome scaffold to deliver MSC-Exos, which had the ability to preserve exosomes for more than 7 days and significantly accelerated the process of cartilage regeneration *in vivo* Chen et al. (2019).

## 4 Conclusion and perspective

MSC-Exos, as a cell-free therapy, provides an advanced strategy for alleviating the progression of OA (Boulestreau et al., 2021; Fan et al., 2022). The role of MSC-Exosomes in chondrocyte regeneration, immunomodulation and ECM balance has been extensively studied. However, current studies on MSC-Exos for the treatment of OA are still in early stages. Most studies are based on small animal models, necessitating validation through large animal models before advancing to clinical research (Yu et al., 2022). Currently, there is great variability in the preparation of MSC-Exos, which may be affected by different MSC sources, culture conditions, and exosomes harvesting strategies (Gimona et al., 2021). Owing to the diverse contents and function of exosomes, it is essential to explore the characterization of MSC-Exos in different subpopulations and accurately determine the content of their cargo, which may alter the impact on the target tissue (Forsberg et al., 2020). Therefore, more attention needs to be paid to make standardized, convenient, and strictly controlled methods in the future. In addition, the shortage and strict selection of MSC donors need to be taken into account. For example, BM-MSCs are difficult to isolate and obtain due to the surprisingly low content (less than 0.01% of the cells in the bone marrow) (Yang et al., 2018). And bone marrow collection is an invasive and painful procedure for donors, which may lead them to abandon donation. As for UC-MSCs, the infectious and familial genetic disease of pregnant woman need to be considered (Tang et al., 2022). So, the challenge of eliminating or inactivating pathogens while retaining the properties of exosomes also needs to be addressed (Burnouf et al., 2019). In order to achieve a therapeutic effect, it is necessary for MSC-Exos to carry bioactive factors like proteins or miRNAs at a sufficient dosage and with functional activity to elicit biological responses in target cells (Toh et al., 2018). However, Chevillet et al. found that most exosomes did not carry biologically significant amounts of miRNAs Chevillet et al. (2014). Therefore, it is particularly important to increase miRNA content by loading methods such as electroporation.

In recent years, although a variety of MSC-Exos engineering strategies have been developed to improve therapeutic efficacy, challenges remain. Large-scale production of MSC-Exos is still a big challenge to be solved for clinical application. And homogenous and high-purity exosomes are hard to obtain by existing time-consuming and low-yield isolation techniques (Charoenviriyakul et al., 2017). Recently, bioreactors or microfluidic platforms have been used to increase the production of exosomes. It was reported that a microfluidic cell culture platform was developed that could harvest large-scale and antigen-modify exosomes in one workflow (Zhao et al., 2019). Furthermore, it should be noted exosomes contain some functional proteins and immune molecules, so the use of engineered exosomes may trigger a strong response by the host immune system and be rapidly eliminated (Lim et al., 2019). Meanwhile, many factors including storage conditions and time, administrate path and dose affect the biological activity and therapeutic efficacy of MSC-Exos.

To our delight, there are several clinical trials underway to evaluate MSC-Exos therapy for OA, and another one has been completed. The current results have shown that the use of MSC-Exos for OA treatment is effective and safe, and has the potential to be an alternative to joint replacement surgery. In summary, MSC-Exos is a promising cell-free therapy for knee OA and deserves more attention.
